# Male alliance behaviour and mating access varies with habitat in a dolphin social network

**DOI:** 10.1038/srep46354

**Published:** 2017-04-13

**Authors:** Richard C. Connor, William R. Cioffi, Srđan Randić, Simon J. Allen, Jana Watson-Capps, Michael Krützen

**Affiliations:** 1 Biology Department, UMASS Dartmouth, North Dartmouth, MA 02747, United States of America; 2 University Program in Ecology, Duke University Marine Lab, Beaufort, NC 28516, United States of America; 3 European Parliament, Bât. PHS, 06A056, 60 rue Wiertz, B-1047 Brussels, Belgium; 4 School of Biological Sciences and Oceans Institute, University of Western Australia, Perth, WA 6009, Australia; 5 Evolutionary Genetics Group, Department of Anthropology, University of Zurich, Winterthurerstrasse 190, CH-8057 Zurich, Switzerland; 6 University of Colorado, BioFrontiers Institute, CU-Boulder, Boulder, CO 80303, United States of America

## Abstract

Within-species variation in social structure has attracted interest recently because of the potential to explore phenotypic plasticity and, specifically, how demographic and ecological variation influence social structure. Populations of bottlenose dolphins (*Tursiops* spp.) vary in male alliance formation, from no alliances to simple pairs to, in Shark Bay, Western Australia, the most complex nested alliances known outside of humans. Examination of ecological contributions to this variation is complicated by differences among populations in other potentially explanatory traits, such as phylogenetic distance, as well as female reproductive schedules, sexual size dimorphism, and body size. Here, we report our discovery of systematic spatial variation in alliance structure, seasonal movements and access to mates within a single continuous social network in the Shark Bay population. Participation in male trios (versus pairs), the sizes of seasonal range shifts and consortship rates all decrease from north to south along the 50 km length of the study area. The southern habitat, characterised by shallow banks and channels, may be marginal relative to the open northern habitat. The discovery of variation in alliance behaviour along a spatial axis within a single population is unprecedented and demonstrates that alliance complexity has an ecological component.

Ecological models of social evolution attempted to explain variation in social and mating systems with key variables such as predation risk and resource distribution^1^,^2^,^3^. Model tests relied on comparative analyses of interspecific and intergeneric variation, based on ‘typical’ species attributes. More recently, the strength of phylogenetic signals in such comparative analyses^4^,^5^ refocused attention on intraspecific variation in social systems, which cannot be attributed to phylogeny^6^,^7^,^8^,^9^,^10^.

While intraspecific variation in social systems is found in a range of taxa, mammals are especially attractive subjects because of their ‘extremely broad range of social systems, behavioural flexibility, brain size and cognitive abilities’^9^. Intraspecific variation in mammalian social systems has been attributed to stochastic demographic effects, local culture and ecology^6^,^7^,^9^.

One component of social behaviour that is more prominent in mammals is the presence of coalitions or alliances within social groups, where two or more individuals cooperate against conspecifics^11^. Within-group alliance relationships may be complex and, hence, relate directly to cognitive abilities and brain size^12^.

The relationship between brain size, life history and complex social relationships has been explored extensively in mammals (e.g. refs 13, 14, 15, 16), as has the relationship between social systems, ecology and demography (e.g. refs 17 and 18). Relatively unexamined, however, is how variation in ecology impacts the complexity of social relationships, such as alliance formation.

Bottlenose dolphins (*Tursiops* spp.) offer a striking example of inter population variation in male alliance behaviour, as illustrated by the contrast between the subtropical embayments of Shark Bay, Western Australia, and Sarasota Bay, Florida, the world’s two longest running dolphin studies^19^. Both populations exhibit fission-fusion grouping patterns with stronger same-sex associations and male ranges that are, on average, larger than female ranges.

In Shark Bay, Indo-Pacific bottlenose dolphins (*T. aduncus*) form an open social network along the 50 km length of the study area (Figs 1 and S1). Males form up to three nested levels of alliances: 2–3 males (=1^st^-order alliances) cooperate to consort individual females; teams of 4–14 males (=2^nd^-order alliances) cooperate in contests with other groups over females, as may teams of 2^nd^-order alliances (=3^rd^-order alliances) that associate at low levels and have generally affiliative relationships^20^,^21^,^22^. Males select 1^st^-order alliance partners almost exclusively from their 2^nd^-order alliance^21^. Male-male associations in 1^st^-order alliances are variable; some are strong and stable, with half-weight association coefficients from 0.80–1.00 (ref. 23, Supplementary Information), lasting up to 20 years^22^, while others are more labile, with changing alliance partnerships between consortships^24^. Within 2^nd^-order alliances, consortship rate is correlated with the stability of individual males’ 1^st^-order alliances, suggesting that dominance relationships may be important in these groups (ref. 22, Supplementary Information).

In contrast to Shark Bay, male bottlenose dolphins (*T. truncatus*) in Sarasota Bay, Florida, form stable pairs, but not trios, to consort females and there is no evidence of higher levels of alliance formation^25^.

Male alliance relationships in Shark Bay are more complex than those in Sarasota, based on the presence of trios instead of pairs, as well as two additional levels of alliance formation (Supplementary Information). Our ability to explore ecological contributions to these differences in alliance complexity is limited by the large number of possible non-ecological confounds. The phylogenetic distance between the two species may impact heavily on differences in alliance formation, but, in this case, there is no reason to invoke non-adaptive explanations (e.g. ref. 4). Connor *et al*.^19^ reviewed inter population variation in reproductive, life history and anatomical traits that would likely impact selection on male alliance formation, including female reproductive schedules (which would affect the operational sex ratio), sexual size dimorphism and body size. Ostensibly, given that bottlenose dolphins are widely distributed, one might be able to identify and compare multiple populations that vary in each of these, as well as ecological factors.

However, with an expansion of the study area in Shark Bay, we discovered systematic spatial variation in alliance behaviour, not only within the Shark Bay population, but also within one continuous social network^26^. We enlarged our study to include twelve 2^nd^-order alliances in order to examine the relationship between 2^nd^-order alliance size and 1^st^-order alliance stability^21^. In a classic case of serendipity, data collected for that study revealed variation among 2^nd^-order alliances in the proportion of 1^st^-order alliances composed of trios versus pairs, the rate that males consort females, and in seasonal range shifts. Here, we present analyses of the proportion of trios, consortship rate and seasonal movements with respect to location and discuss ecological hypotheses that might help explain our results.

## Results

### Trio formation

We confirmed 293 mating season consortships (mean over alliances = 24.4, range = 4–72). The overall rate of trio formation was high at 0.85 (250/293). The seven northern 2^nd^-order alliances formed trios significantly more than the five southern 2^nd^-order alliances (GLM, z = −8.03, p < 0.001). The northern 2^nd^-order alliances used trios almost exclusively (0.98, 227/231), while the proportion of trios in the south was 0.37 (23/62). NW-SE axis distance was also a significant predictor for alliance trio formation in a binomial GLM (Fig. 2; GLM, z = −7.41, p < 0.001). Trio use increased sharply just north of the southern habitat (Fig. 2). See Table S1a for model coefficients.

Group size may impact social structure (e.g. refs 7 and 27) and the largest 2^nd^-order alliances were in the northern part of the study area. However, the small northern 2^nd^-order alliances (Table S2, alliance 3 & 5) also formed trios almost exclusively. Further, by 2010, a southern group grew from eight members to become the third 2^nd^-order alliance to achieve a size of 14 members^22^, but continued to form pairs and trios through 2014 (unpub. data). Alliance 8 (Table S2) was an outlier among the southern 2^nd^-order alliances as they consorted females in two completely stable trios. One southern group (Table S2, alliance 9) was the only 2^nd^-order alliance to employ pairs exclusively.

### Consortship rate

A total of 2,146 observations were scored for consortships (mean over alliances = 214.6, range = 37–660). The overall consortship rate was approximately 0.58 (1,237/2,146). The northern (n = 6) 2^nd^-order alliances had higher consortship rates than southern (n = 4) 2^nd^-order alliances (GLM, z = −8.44, p < 0.001), with a mean difference of approximately 0.27 (see Methods). NW-SE axis distance was also a significant negative predictor for 2^nd^-order alliance consortship rate (Fig. 2; GLM, n = 10, z = −7.34, p < 0.001). Consortship rates appeared to increase relatively smoothly from the SE to the NW (Fig. 2; see Table S1 for model coefficients).

We compared the maximum individual consortship rates for each 2^nd^-order alliance and found a similar relationship with the NW-SE axis position significantly predicting consortship rate (Fig. 3; GLM, n = 10, z = −3.26, p = 0.001). We also calculated consortship rates for the maximum consorting dyad in each 2^nd^-order alliance and, again, found a similar relationship (Fig. 3; GLM, n = 10, z = −4.47, p < 0.001; see Table S3 for model coefficients). The same analyses for individuals with low consortship rates in each 2^nd^-order alliance demonstrate that the NW to SE decline in consortship rates is general and not due, for example, to exceptionally high rates among a few individuals in northern 2^nd^-order alliances (Supplementary Information).

### Adjusted consortship rate

When trio formation was accounted for in the adjusted consortship rate (that controls for pair vs trio formation, see Methods, Fig. 4), a smaller, but still significant, effect in the same direction was found between northern and southern alliances (GLM, n = 10, z = −2.12, p = 0.034) and along the NW-SE axis (GLM, n = 10, z = −2.17, p = 0.030), with consortship rates increasing in the further NW alliances (Fig. 2). The overall adjusted consortship rate was lower at 0.40 (430/1,073), with a narrower range from 0.26 (16/62) to 0.54 (116/330). See Table S1 for model coefficients.

### Range shifts

The seven northern alliances all showed seasonal range shifts in a south-easterly direction during the mating season (Fig. 5). There was greater variation in the direction of range shifts of the five southern alliances and these shifts tended to be smaller (northern mean ± SD = 6.7 ± 5.7 km; southern mean ± SD = 1.9 ± 0.98 km), but the difference was not significant (n = 12, t = 2.19, p = 0.068).

NW-SE axis position was a significant predictor of range shift distance (linear regression, adj. R^2^ = 0.62, t = −4.36, p = 0.0014). This result was likely driven by the three northernmost alliances, which had the largest range shifts observed (Fig. 5). See Table S4 for model coefficients.

## Discussion

We have shown that 1^st^-order alliance size, consortship rate and seasonal ranging of male bottlenose dolphins vary systematically along the Peron Peninsula in the eastern gulf of Shark Bay. This variation demands an ecological explanation, rather than one based on stochastic demographic or cultural variation, as is sometimes found among different primate groups in the same population (refs 1 and 2, Supplementary Information) or individual dolphins in Shark Bay^28^. We know of no model of random demographic or cultural variation that could yield systematic spatial variation in alliance structure and the rate that males consort females and, therefore, assume a model based on the economics of male mate acquisition and defence under varying ecological conditions.

Perhaps unique among mammals is our discovery of systematic spatial variation in alliance behaviour within a single social network^26^. In fact, systematic spatial variation in any major component of social organisation is rare within social networks (for an example in feral horses, see ref. 29) compared to that between geographically separated groups. In a recent review, Kappeler *et al*.^9^ stated that ‘with increasing geographical distance among sub-populations, ecological factors are more likely to vary in ways that influence behavioural variation among populations. These factors include population density, predation risk and food availability’.

As population density increases, so will the rate individuals encounter each other in competitive circumstances, favouring the formation of alliances or larger alliances^30^,^31^. A 20-year study revealed temporal, but not spatial, changes in alliance formation within a population of cheetahs (*Acinonyx jubatus*)^32^. The survival of male cheetahs in coalitions was higher when there were more coalitions in the area, and lower than that of singletons when there were low numbers of coalitions^32^. In accord with the encounter rate model, the authors attributed this to the ability of singletons to hold territories when competing with high or low numbers of coalitions.

The lower proportion of trios in the southern habitat could be explained by higher encounter rates with receptive females, reducing the need for larger alliances. This hypothesis is refuted by the lower consortship rates in the south, which indicate reduced access to females. Since males go where females are (e.g. ref. 33), the lower consortship rate and proportion of trios in the southern part of the habitat may reflect a lower density of dolphins in that area (P. Berggren unpubl. data). Population density differences should reflect differences in the abundance of the resources upon which dolphins rely. The possibility of significant resource variation along the NW to SE axis is suggested by the strong salinity gradient in Shark Bay, with hypersaline waters in the innermost basins of Hamelin Pool and L’haridon Bight^34^. The southern part of our study area abuts the depauperate, hypersaline waters of L’haridon Bight to the south and may be marginal habitat for bottlenose dolphins in Shark Bay. It is worth noting here that the seasonal southward shift of the northern alliances, around the onset of the mating season, falls short of the southern habitat. An interesting question is whether the northern males are simply following females (that are, in turn, responding to changes in food or predator distribution) and/or are being ‘pushed’ by other, possibly larger alliances (see Fig. 5), as Wilson *et al*.^35^ suggested to explain a similar pattern of dolphin group movements in the Moray Firth, Scotland. Regardless, there appears to be a mating season ‘compaction’ of alliances that should increase the encounter rate and, hence, competition for females.

Resource distribution can also influence group size directly. For example, a greater abundance of schooling fish in open habitat could favour trios by reducing grouping costs or enhancing the benefits if the dolphins have a greater ability to detect prey or forage cooperatively on fish schools.

Encounter rates, and thus alliance formation, may be impacted not only by population density, but also ranging patterns and the distances over which individuals can detect each other^30^. The latter is well illustrated by African lion (*Panthera leo*) prides, which are alliances of related females. Lions live at high density, but so do solitary felids. Critically, lions also feed on large carcasses in open habitat, which last for some time and would attract rival groups from greater distances, effectively increasing encounter rates compared to more concealed felids living at a similar population density (ref. 36, see also ref. 32). Similarly, the sharp increase in trio formation in Shark Bay (Fig. 2) coincides with the abrupt change to open habitat, where dolphins may be able to detect rivals at greater distances, as vocalisations are likely to travel farther^37^. Differences in day and home ranges between the northern open and southern subdivided habitat could also impact encounter rates (see refs 38, 39, 40).

A higher predation risk is associated with the transition from uni- to multi-male social structure in some primates, but not others (reviewed in ref. 6). In Shark Bay, areas with greater predation risk could favour trios over pairs^19^, resulting in higher unadjusted consortship rates (see Fig. 4). The predation risk model encounters difficulty when we consider results based on the consortship rates of the top male and top pair for each 2^nd^-order alliance. If dominance within 2^nd^-order alliances is important and there is no difference in habitat quality along the length of the study area, one would expect the top males in each group to have similar consortship rates (the inter-group differences would be due to lower consortship rates among low ranking members of southern 2^nd^-order alliances, see Fig. 4). However, this is not the case.

The predation risk model can still be rescued if we include ‘ownership’ in our model, where pairs or trios in the same alliance do not (or rarely) contest each other for females. Such an ownership rule has been reported in male lions with oestrus females^36^, and appears to apply to captured fish in the Shark Bay dolphin population^19^, so it is not unreasonable to imagine that it might extend to consortships (here, we are assuming that the relationship between 1^st^-order alliance stability and consortship rate does not imply dominance or that dominance is manifest in other ways, such as mating access to females being consorted by other 1^st^-order alliances in a male’s 2^nd^-order alliance). However, the results from the adjusted consortship rate analysis, which controls for trio vs pair formation, are incompatible with a predation risk model that includes ownership.

The bottlenose dolphins inhabiting waters off the east side of Peron Peninsula in Shark Bay live in an open social network with nested male alliances^22^,^26^. The convergence between humans and dolphins in nested male alliances with context-dependent interactions among particular individuals and alliances has contributed to our understanding of the ‘social brain’ hypothesis^16^,^41^,^42^. Although some populations of bottlenose dolphins have simpler male alliances, they also differ in other variables that make it difficult to determine if there are ecological contributions to population differences in dolphin alliance behaviour. Here, we have shown that the Shark Bay dolphin alliances vary systematically along a spatial axis in alliance structure, consortship rates and ranging behaviour. Hypotheses based on predation risk and food distribution can explain the shift from pairs to trios, but not the change in consortship rate. However, alliance size and consortship rate can be explained by factors that impact the rate at which males encounter each other in competition over females, such as population density (and possibly ranging patterns) and detection distance. Our discovery of systematic spatial variation in alliance behaviour demonstrates that alliance complexity has an ecological component.

## Methods

### Study subjects and site

**Figure 1:**
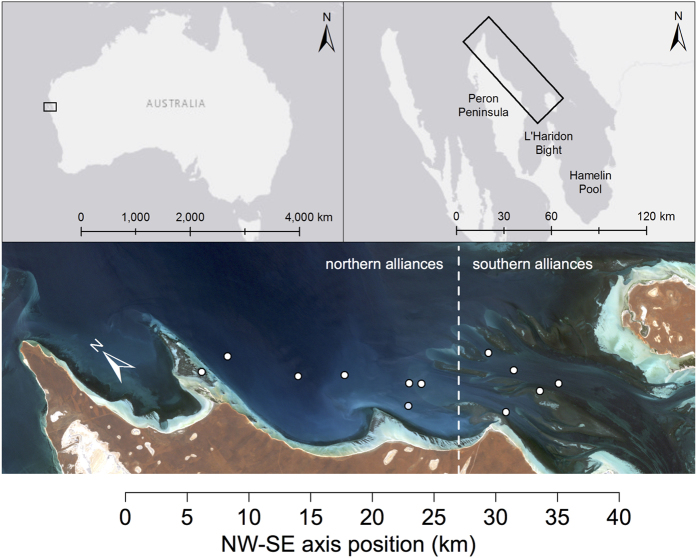
The study site in waters off the east side of Peron Peninsula, which bisects Shark Bay, Western Australia. Centroids for seven northern 2^nd^-order alliances, which occupy relatively open habitat, are shown divided from the five southern 2^nd^-order alliances, which occupy habitat subdivided by shallow banks and channels. Landsat 7 ETM+ imagery of Shark Bay courtesy of the U.S. Geological Survey.

**Figure 2:**
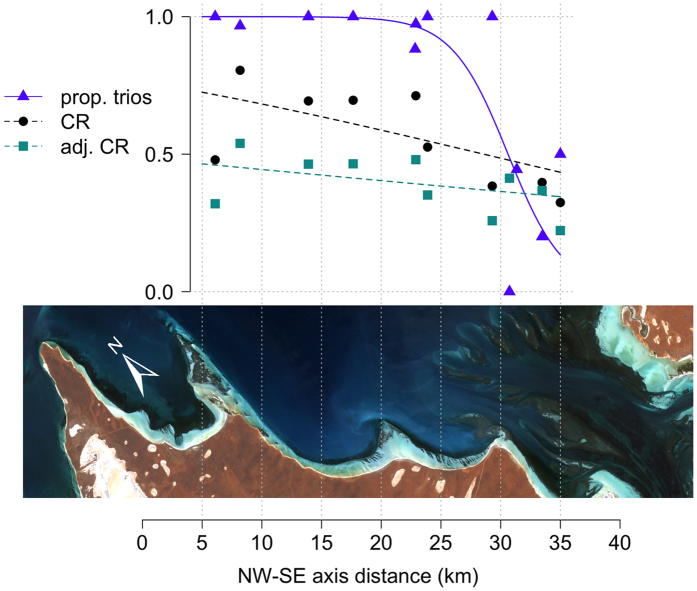
The proportion of trios (triangles), consortship rate (circles), and adjusted consortship rate (squares) in 2^nd^-order alliances decreases in a SE direction across the study area/two habitats. Fitted logistic curves are shown from generalized linear models. prop. trios = proportion trios, CR = consortship rate. Landsat 7 ETM+ imagery of Shark Bay courtesy of the U.S. Geological Survey.

**Figure 3:**
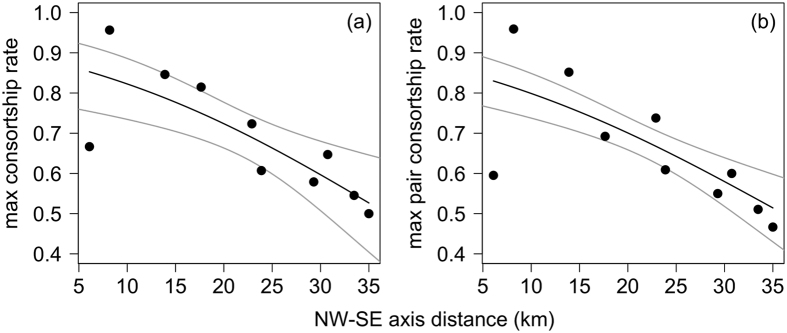
For each of the 12 2^nd^-order alliances, the (**a**) maximum consortship rate (CR) for an individual, and the (**b**) summed consortship rates of the strongest bonded pair with highest consortship rates are plotted along the NW-SE axis. Grey lines show 95% confidence intervals.

From 2001–2006, we studied the behaviour and ranging patterns of 121 adult male Indo-Pacific bottlenose dolphins (*Tursiops aduncus*) in waters off the east side of Peron Peninsula, which bisects Shark Bay (Fig. 1; refs 21 and 26). These males associated in 12 2^nd^-order alliances and five ‘lone trios’. The lone trios were primarily very old males, whose former 2^nd^-order alliance partners had disappeared^21^. Here, we focus on data from the 12 2^nd^-order alliances. Alliance ranges overlapped extensively, but the ranges of alliances at either end of the study area did not overlap (ref. 26, Fig. S1).

The study area is divided into two quite distinct habitats. The southern part of the study area is characterised by ‘offshore’ shallow banks (clearly visible in Fig. 1) that are subdivided by deeper channels, while open embayment plains without shallow banks characterise the northern part of the study area.

### Field observations

Male association, behavioural and ranging data were collected through dolphin group surveys and focal follows during the same five-month field season for six years (2001–2006, from July through November). This period includes the first three months of the peak-breeding season (Austral spring, Sep-Nov), when conceptions (as indicated by births, given a 12-month gestation period), the duration of female ‘attractive periods’ (based on the duration of single and consecutive consortships) and 1^st^-order alliance stability all increase sharply^22^,^43^,^44^,^45^.

Surveys represent brief encounters (at least five minutes) with dolphin groups during which we record a variety of data, including time and GPS location, as well as group composition, based on photographic identification^46^. A group was defined as all individuals that were observed together based on a 10 m ‘chain rule’^23^.

Focal alliance follows ranged in duration from 1 to 8 hours. During follows, we kept a continuous record of behaviour and group membership, with a focus on documenting consortships, following Connor *et al*. (ref. 24, Supplementary Information).

### Analysis

We investigated the influence of 2^nd^-order alliance location within the habitat on three main response variables: proportion of trios, consortship rate and seasonal range shift using generalized linear models (GLMs) and linear models (LMs) implemented in R^47^. Tests on proportion of trios and consortship rate reported here were performed using data collected during the peak reproductive season (September – November; for data see Tables S5 and 6). Non-mating season results are presented in the Supplementary Information. We performed statistical analyses at the level of the 2^nd^-order alliances to control for possible group level behavioural effects. Proportion of trios and consortship rate were both conceived of as binomially distributed random variables, where we assumed an underlying probability of consortships and forming trios in those consortships for each 2^nd^-order alliance. Range shifts were analysed using ordinary least squares regression, because errors were judged normally distributed. Heteroscedasticity of errors were assessed for all models by inspection and we used a significance level of alpha = 0.05.

We calculated 2^nd^-order alliance position from GPS location data obtained during the surveys. Positions were projected into Universal Transverse Mercator (zone 49S, WGS84 datum) in ArcGIS 10.2.2 (ESRI 2014). Each location represented a sighting of an individual on a given day. Only the first sighting per individual per day was used to decrease auto-correlation. These locations were used to calculate home range centroids for each 2^nd^-order alliance, representing the geometric centre of all group members during the study period. In addition to this overall centroid, we calculated season-specific centroids including only sightings during the pre-mating season (July–August) and the mating season (September–November).

The home range centroids of five of the 2^nd^-order alliances were south of the northern edge of the shallow banks and channels, while the centroids of the other seven 2^nd^-order alliances were north of the banks and channels (Fig. 1). This provided a categorical predictor variable of 2^nd^-order alliance position.

There is, however, continuous range overlap among 2^nd^-order alliances in Shark Bay and those whose centroids were located near the line in Fig. 1 ranged in both habitat types (see ref. 26, Fig. S1). Therefore, we also calculated a continuous predictor variable representing habitat position for each 2^nd^-order alliance. Second-order alliance centroids lay roughly along a north-western/south-eastern (NW-SE) axis, following the coast of the Peron Peninsula. A least squares best-fit line was obtained for the centroids and used as a new rotated axis to calculate the distance of each centroid along the line with the value increasing in the SE direction. This NW-SE axis position (measured in km) provided a single continuous variable to describe the centre of each 2^nd^-order alliance’s range.

#### Trio formation

The proportion of trios for each 2^nd^-order alliance was calculated as the number of consortships by trios divided by the total number of consortships (binomial trials).

#### Consortship rate

Consortship rates for each male were calculated as the number of days in a consortship (successes) divided by the total number of days that animal was observed over the entire study period (binomial trials)^30^. Individual consortship rates for each member of a 2^nd^-order alliance were summed to create an overall consortship rate. Two 2^nd^-order alliances were composed of young members who were just starting to display reproductive behaviours during the study period. To minimize possible age effects, these alliances were removed from the analyses of consortship rate, leaving 10 alliances.

Within 2^nd^-order alliances, male consortship rate correlates with 1^st^-order alliance stability (refs 22 and 24, Supplementary Information), suggesting that dominance relationships may be important in these groups. If there is no effect of habitat, then the consortship rate of the top ranking males of each 2^nd^-order alliance should not vary systematically along the NW-SE axis. We therefore examined the relationship between position on the NW-SE axis and the highest individual consortship rate for each 2^nd^-order alliance and the average consortship rate of the strongest-bond pair in each group.

#### Adjusted consortship rates

**Figure 4:**
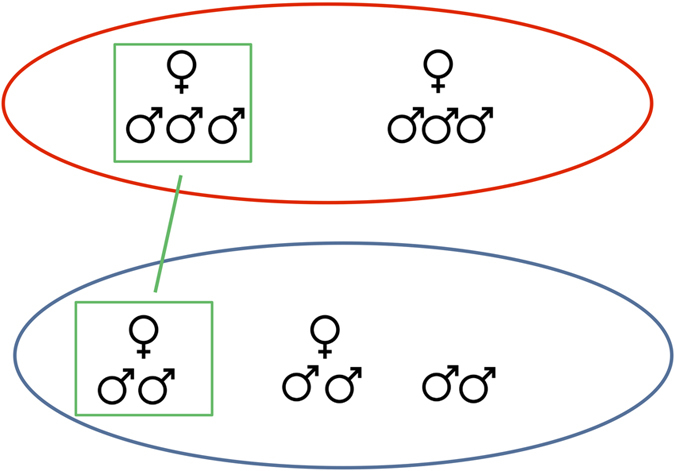
Two 2^nd^-order alliances whose males form trios (top) or pairs (bottom). If two females are available for each 2^nd^-order alliance, each male in the trio-forming 2^nd^-order alliance will be scored in a consortship, but a consortship will be scored for only four of six males in the pair-forming 2^nd^-order alliance. This will lower the overall consortship rate for the pair-forming 2^nd^-order alliance relative to the trio-forming group.

**Figure 5:**
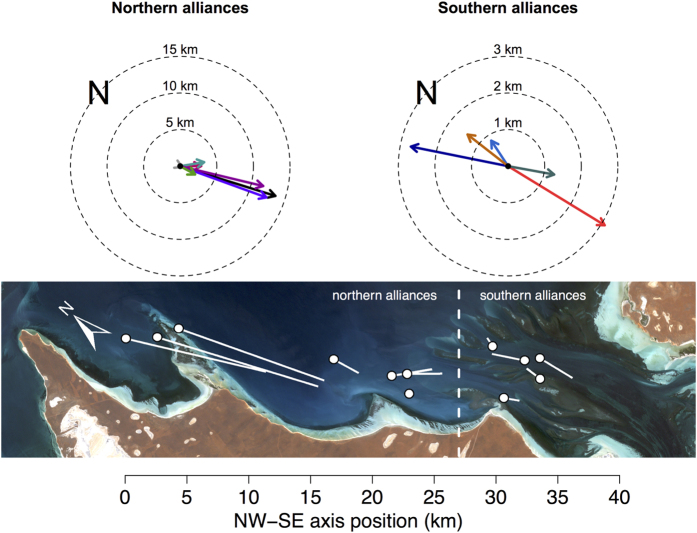
Seasonal range shifts for each 2^nd^-order alliance were calculated as the distance and direction from each alliance’s winter (July–August) range centroid (dots) to their spring/summer (September–November) range centroid (end of line). Landsat 7 ETM+ imagery of Shark Bay courtesy of the U.S. Geological Survey.

Differences in consortship rate may be influenced by the size of the consorting alliance (pair or trio). Consider a 2^nd^-order alliance of six males in the north that forms trios only and one in the south that forms pairs only, that only two females are available to each 2^nd^-order alliance, and that males search randomly for females in pairs or trios. When both females are being consorted all the males in the northern alliance, but only four of the six males in the southern group, will be scored in a consortship (Fig. 4). Thus, on average, males in the south will suffer a lower consortship rate. To control for this possible confound, we calculated an adjusted consortship rate where each consort day in a pair counted as 1/2 and each consort day in a trio counted as 1/3. Total days in consortships, representing total binomial trials, were multiplied by 0.5, representing the maximum possible value if a male had been in a pair for all of his consortships. This adjusted consortship rate was compared for the northernmost and southernmost alliances using a binomial GLM, as above. Since the above multiplication sometimes produced non-integers, these were rounded before being entered into the binomial model.

#### Range shifts

To estimate seasonal shifts, the magnitude (in km) and compass direction (azimuth) of the vector connecting the seasonal 2^nd^-order alliance centroids were calculated^48^.

## Additional Information

**How to cite this article**: Connor, R. C. *et al*. Male alliance behaviour and mating access varies with habitat in a dolphin social network. *Sci. Rep.* **7**, 46354; doi: 10.1038/srep46354 (2017).

**Publisher's note:** Springer Nature remains neutral with regard to jurisdictional claims in published maps and institutional affiliations.

## Supplementary Material

Supplementary Information
