# Supplementary material for: Male alliance behaviour and mating access varies with habitat in a dolphin social network
Source: Sci Rep. 2017 Apr 13;7:46354. doi: 10.1038/srep46354 (PMC5390316; doi:10.1038/srep46354)
Supplement: Supplementary Information [file srep46354-s1.pdf]

Supplementary Information

Male alliance behaviour and mating access varies with habitat in a dolphin social network

Connor, Cioffi, Randić, Allen, Watson-Capps, Krützen

Supplementary Notes

Male alliances in Shark Bay and Sarasota.

Trios of allied male dolphins are much more common than pairs in Shark Bay [1]. Second-order alliances, teams of 4-14 males that form 1st-order alliances and cooperate to capture females from other alliances or to defend against such attacks, are considered the core male social unit in Shark Bay and may endure for at least two decades [2]. Second-order alliance membership changes slowly, mostly through attrition, as members age and die, but even old, well-established groups may occasionally add a new member. Dominance relationships among male bottlenose dolphins have been demonstrated in captivity [3], but not in the wild. Our discovery of a correlation between individual male consortship rates and 1st-order alliance stability in 12 2nd-order alliances provides the strongest, although indirect, evidence of dominance relationships in wild bottlenose dolphins [2]. It is not clear if Sarasota males employ aggression to establish and maintain consortships, as occurs commonly in Shark Bay [4-5].

Half-weight association coefficient.

At sea, we are more likely to see one of a pair of dolphins when they are apart, in two different groups, than together in one group. Under these conditions the half-weight association coefficient performs well [6], so many dolphin researchers have adopted this measure (perhaps more so now to enable comparison with earlier studies). The half-weight coefficient is defined as $2N_{ab}/(N_a + N_b)$, where N_{ab} is the number of groups in which A and B are found together and N_a and N_b are the total number of group sightings for A and B, respectively. This equation yields association coefficient values ranging from 0 (for two individuals that are never sighted together in groups) to 1 (for individuals that are always sighted together).

Alliance stability index.

Connor et al. [7] calculated an 'alliance stability' index for individual males within a single, large 14-member second-order alliance as follows: [1 - (number of different 1st-order alliances/number consortships)]. This value will be zero for a male that has a different set of 1st-order alliance partners for each consortship and will approach one (with increasing consortship sample size) for males that keep the same partners. This index correlated with individual consortship rates within the 14-member alliance and was later found to correlate with consortship rates in 12 2nd-order-alliances ranging in size from 6-14 males [2].

Alliance complexity.

Alliances and coalitions within a group or social network are more complex than simple 'us against them' between-group conflicts because they include competition for alliance partners based on affiliative interactions, and context dependent interactions, where friends in one social context may become foes in another [8-11]. However, even within-group alliances need not be complex if, for example, they are based entirely on close kinship [11]. Indeed, one study failed to find a relationship between brain size and coalitions in primates [12]. In stable Shark Bay trios, one pair of males more often associates and synchronises their swimming than either does with the third member or 'odd male out', but on a given day the odd male out may shift [13-14]. Such complexity is not possible in pairs. Context dependent and affiliative interactions have been observed in all three levels of Shark Bay male dolphin alliances [2]. Additional alliance levels should increase the complexity of individual decisions because conflicts at one level can impact alliances at another level. For example, a dolphin trio that evicts one of their members to reduce the costs of sharing consorted females should be more vulnerable in 2nd -order alliance contests [2, 9-10].

Supplementary Results

Non-mating season

We found that NW-SE axis distance still significantly predicted proportion of trios, consortship rate, and adjusted consortship rate during the non-mating season (July-August) (Table S1b). Consortship rate and adjusted consortship rate both tended to

decrease for 2nd-order alliances in the non-mating season (Fig. S2) with mean differences of 0.26 and 0.19 respectively ($n = 10$ in both cases). In contrast to consortship rate, proportion of trios in consortships did not show a consistent trend between the seasons (Fig. S2) with a mean difference of 0.03 ($n = 12$).

Low consortship rate variation along the penninsula

In the main text we report a significant relationship between NW-SE axis position and both the maximum individual and maximum consorting dyad consorthip rates for each 2nd-order alliance. We also compared the minimum individual consortship rates for each 2nd-order alliance and found a similar relationship with the NW-SE axis position significantly predicting consortship rate (Fig. S3; GLM, $n = 10$, $z = -2.16$, $p = 0.031$). Since the lowest consortship rate in three groups were outliers (Fig. S3), we also calculated consortship rates for the 2nd lowest consorting individual (Fig. S3; GLM, $n = 10$, $z = -3.00$, $p = 0.027$) and the 2nd and 3rd lowest individuals together (Fig. S3; GLM, $n = 10$, $z = -3.62$, $p < 0.001$) and found similar trends. Together, the analyses of individuals with high and low consortship rates in each 2nd-order alliance demonstrate that the NW-SE decline in consortship rate is robust and not due to a few extreme values in northern or southern groups.

Supplementary Discussion

Individual foraging tactics in Shark Bay

Whereas foraging cultures may vary across groups and populations of primates and cetaceans [15-17], in marine mammals it is also common to find different foraging tactics among individuals that occupy the same area. Some female and male dolphins in Shark Bay with extensively overlapping ranges employ different foraging tactics. Some of these individual foraging tactics are quite striking, including 'sponge-carrying', 'beaching', 'shelling' and 'kerplunking' [18-23].

Supplementary Methods

The study subjects and site.

In Shark Bay, over 1500 individually identified dolphins have been observed since the mid-1980s [reviewed in 2]. The dolphins exhibit a highly dynamic fission-fusion grouping pattern in an open social network: a mosaic of overlapping male and female ranges extends along the 50km length of the study area, but dolphins in the northern and southernmost parts of the study area do not overlap (Fig. S1). Females typically conceive first at age 11 and give birth at age 12 to a single infant that is dependent for 3-5 years. Most males are members of 2nd-order alliances and begin consorting adult females by age 13-14. Some males and females live into their 40s.

Documenting Consortships.

Males cooperate in pairs and trios to sequester individual females for periods of minutes to weeks. Defining criteria are outlined in Connor et al. [7], including an association lasting one or more hours, or on multiple surveys spanning one or more hours, a capture of the female by the males, the female attempting to escape (bolting), or aggression by the males toward the female (head jerks, charge or the males producing the 'pop' vocalization). Aggression is observed in about half of consortships. Males almost always consort females with members of their 2nd -order alliance; in about 3% (15 of 496 consorthips), 1st-order alliances contained members of two 2nd-order alliances [1]). By definition, membership is stable for the duration of the consortship. If we find the same trio with a different female the next day or even later on the same day, or there is a change in trio composition, a new consortship is recorded.

Non-mating season vs mating season consortship rates.

Our analysis of consortship rates and trio formation focused on the mating season (September-November) because that is the period during which the majority of births and conceptions occur [24]. Consortships are longer during the mating season [25] and, in this study, consortship rates were higher in the mating compared to the non-mating season. Females that are consorted and conceive during the mating season (based on birth records) are often consorted for short periods during the preceding non-mating season [25]. Thus, females consorted prior to the mating season might not be contested as much as females during the mating

season. For this analysis, we decided, *a priori*, to focus on mating season consortship rates, as that should be the period of greatest intensity of competition for females and where a difference in consortship rates would be of greatest consequence. Combining the periods could potentially obscure important mating season phenomena. This concern was motivated by results from other taxa showing that male mating access may change based on the proximity to female conception. For example, during the period of maximal estrus swelling period in chimpanzees (*Pan troglodytes*), mating and mate guarding increase [26-28], with the greatest degree of dominant possessive behaviour occuring in the few days of maximal fertility, so the extent to which dominant males monopolize females would be obscured by observing male mating rates over a longer period.

Supplementary Figures

Supplementary Figure S1. Ranges (Minimum convex polygon, MCP) of the 12 2nd - order alliances and 5 'lone trios' studied from 2001-2006 (see text). Reprinted from [28], the map was created in ArcGIS. Lone trios (size 3) were excluded from analyses in this paper.

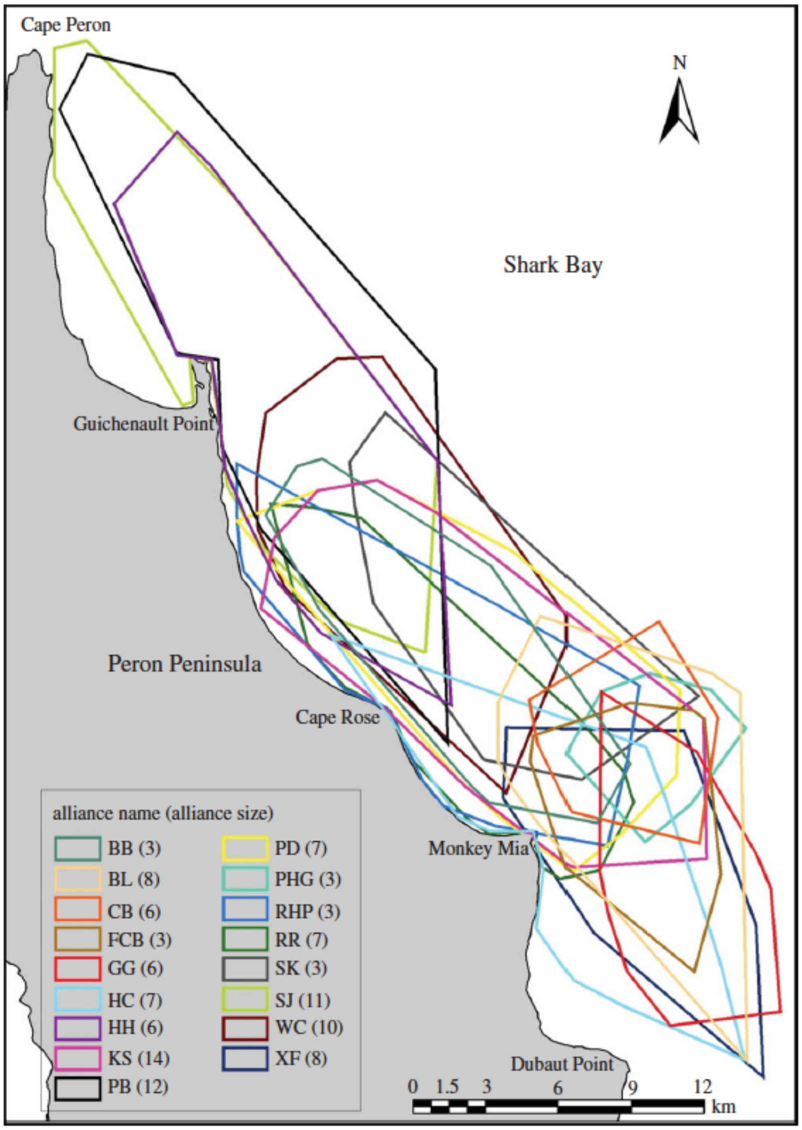

Supplementary Figure S2. Comparison of 2nd-order alliance consortship rate (a), proportion of trios (b), and adjusted consortship rate (c) between the mating and non-mating seasons.

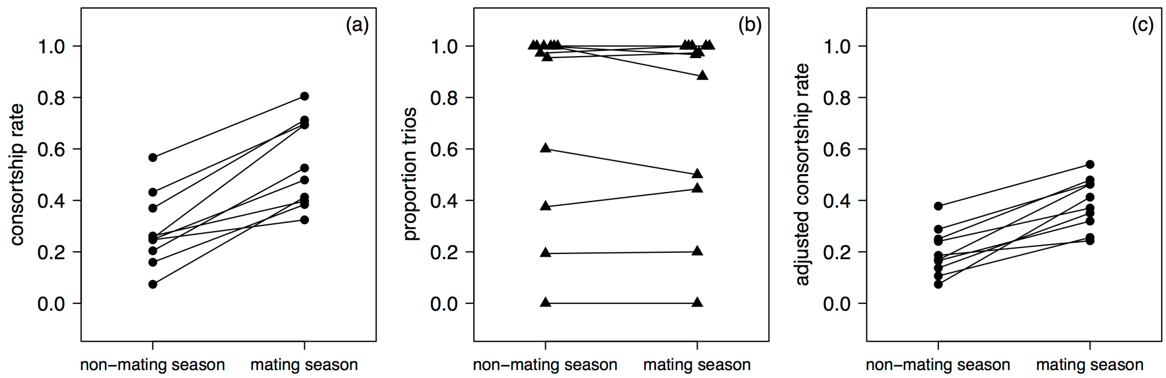

Supplementary Figure S3. For each of the ten 2nd-order alliances, the (a) lowest consortship rate (CR) for an individual, (b) the 2nd lowest consortship rate (CR), and (b) the summed consortship rate of the 2nd and 3rd lowest consorting individuals are plotted along the NW-SE axis. Grey lines show 95% confidence intervals.

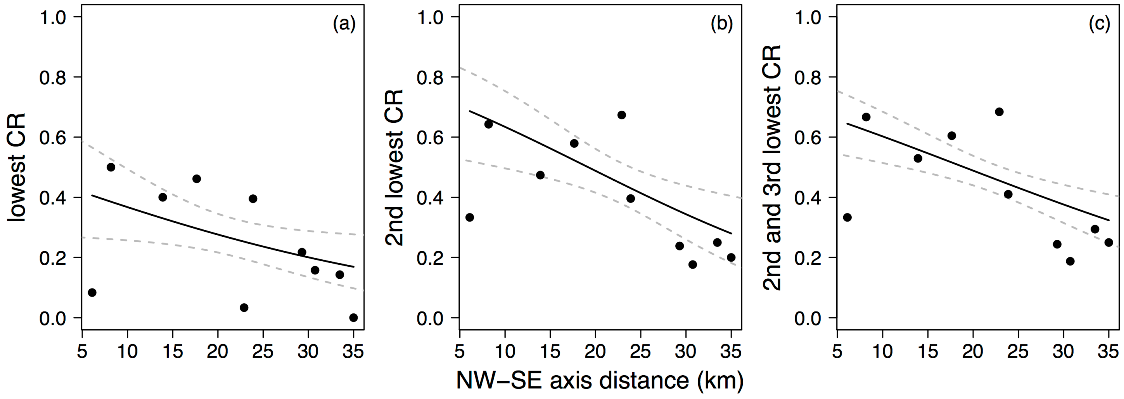

Supplementary Tables

Supplementary Table S1a. Regression summaries for binomial generalized linear models of the proportion trios, consortship rate (CR), and adjusted consortship rate during the mating season (September to Novemeber).

Model	coefficient	estimate	*SE*	*z*	*p*
Proportion trios	Intercept	13.05	1.68	7.76	< 0.001
	NW-SE pos.	-0.426	0.0575	-7.41	< 0.001
CR	Intercept	1.176	0.128	9.21	< 0.001
	NW-SE pos.	-0.0412	0.0056	-7.34	< 0.001
Adjusted CR	Intercept	-0.0579	0.170	-0.340	0.73
	NW-SE pos.	-0.0166	0.00767	-2.17	0.030

Supplementary Table S1b. Regression summaries for binomial generalized linear models of the proportion of trios, consortship rate (CR), and adjusted consortship rate during the non-mating season (July to August).

Model	coefficient	estimate	*SE*	*z*	*p*
Proportion trios	Intercept	15.42	1.65	9.34	<0.001
	NW-SE pos.	-0.49	0.052	-9.25	<0.001
CR	Intercept	-0.066	0.14	-0.47	0.64
	NW-SE pos.	-0.042	0.0058	-7.19	<0.001
Adjusted CR	Intercept	-0.903	0.222	-4.06	<0.001
	NW-SE pos.	-0.024	0.0092	-2.66	<0.01

Supplementary Table S2. Centroid positions for each second order alliance were calculated in Universal Transverse Mercator (UTM) zone 49S coordinates for the entire study period and by season. NW-SE axis position measured in meters was calculatedalong a least squares fit line of all sightings for the study period. Alliances (AL) were also coded as lying in the northern open habitat (N) or southern subdivided habitat (S).

Al	Al size	Centroid UTM-X	Centroid UTM-Y	Centroid Jul-Aug UTM-X	Centroid Jul-Aug UTM-Y	Centroid Sep-Nov UTM-X	Centroid Sep-Nov UTM-Y	NW-SE axis position (meters)	N or S
1	11	760251.3009	7164523.807	757425.0328	7170178.726	763035.5805	7159961.689	6093.651	N
2	12	762559.9705	7163797.015	760912.6621	7167572.927	765667.4801	7155985.065	8178.503	N
3	6	765237.6738	7158510.893	759226.4739	7168372.596	764961.1306	7156014.789	13898.67	N
4	10	767828.8241	7155799.863	767459.4463	7156595.109	767989.2248	7154331.481	17647.17	N
5	7	769522.2439	7150340.043	769468.6108	7150159.916	769553.0859	7149895.986	22837.84	N
6	7	770888.8607	7151494.16	769617.0257	7152200.611	772217.3778	7150147.382	22894.52	N
7	14	771513.5676	7150727.507	770567.4603	7151395.478	772502.5477	7149289.373	23882.27	N
8	6	776966.186	7148350.622	776909.6097	7147791.217	777116.4884	7148601.413	29297.03	S
9	7	774486.7166	7144175.595	774367.6597	7144279.905	775044.4349	7143194.94	30740.82	S
10	8	777332.5763	7145913.908	777788.7222	7145097.354	776398.4907	7147422.794	31353.09	S
11	8	777553.3071	7143261.654	777512.8048	7143145.759	777407.6927	7144547.268	33471.88	S
12	6	779009.2446	7142518.511	778731.1651	7144300.137	779310.7058	7141260.952	34998.33	S

Supplementary Table S3. Regression summaries for lowest (1st, 2nd, and 2nd and 3rd lowest) and highest (maximum and maximum pair) individual consortship rates (CR) in each 2nd-order alliance during the mating season (September to November).

Model	coefficient	estimate	*SE*	*z*	*p*
minimum CR	Intercept	-0.124	0.434	-0.285	0.78
	NW-SE pos.	-0.042	0.019	-2.16	0.031
2nd Lowest CR	Intercept	1.147	0.472	2.43	0.015
	NW-SE pos.	-0.060	0.020	-3.00	0.0027
2nd and 3rd Lowest CR	Intercept	0.876	0.300	2.93	0.0034
	NW-SE pos.	-0.046	0.0127	-3.62	< 0.001
Maximum CR	Intercept	2.11	0.423	4.98	< 0.001
	NW-SE pos.	-0.057	0.0175	-3.26	0.0011
Maximum pair CR	Intercept	1.908	0.284	6.73	< 0.001
	NW-SE pos.	-0.0529	0.0118	-4.47	< 0.001

Supplementary Table S4. Regression summary for range shifts between the non-mating season (July to Agust) and the mating season (September to Novemeber).

Model	coefficient	estimate	*SE*	*t*	*p*
Range shift	Intercept	14000	2310	6.05	< 0.001
	NW-SE pos.	-407.49	93.5	-4.36	0.0014

Supplementary Table S5. Raw counts of mating season days (September-November) sighted in and out of consortships for each second order alliance and number of consortships by trios and pairs. Alliances 5 and 10 were omitted from the consortship analysis to control for age effects because they consisted of young males during the study period.

Alliance	Days in consortship	Days not in consortship	Trios	Pairs
1	93	101	16	0
2	165	40	29	1
3	95	42	20	0
4	158	69	38	0
5			15	2
6	213	86	37	1
7	347	313	72	0
8	48	77	13	0
9	52	74	0	16
10			4	5
11	54	82	4	16
12	12	25	2	2

Supplementary Table S6. Raw counts of days sighted in and out of consortships for lowest and highest consorting dolphins in each 2nd-order alliance during the mating season (September-November). Alliances 5 and 10 were omitted from consortship rate analysis to control for age effects because they consisted of young males during the study period

Alliance	min individual days in consortship	min individual days not in consortship	2nd lowest days in consortship	2nd lowest days not in consortship	2nd and 3rd lowest days in consortship	2nd and 3rd lowest Days not in consortship
1	1	11	2	4	9	18
2	9	9	9	5	18	9
3	8	12	9	10	18	16
4	6	7	11	8	26	17
5						
6	1	29	33	16	65	30
7	17	26	19	29	41	59
8	5	18	5	16	10	31
9	3	16	3	14	6	26
10						
11	2	12	4	12	10	24
12	0	4	1	4	3	9

Supplementary References

1 Connor, R.C., Watson-Capps, J.J., Sherwin, W.S. & Krützen, M. 2011 New levels of complexity in the male alliance networks of Indian Ocean bottlenose dolphins (*Tursiops* sp.). *Biol. Lett.* **7**, 623-626. (DOI: 10.1098/rsbl.2010.0852)

2 Connor, R.C. & Krützen, M. 2015 Male dolphin alliances in Shark Bay: changing perspectives in a 30-year study. *Anim. Behav.* **103**, 223-235.

3 Samuels, A. & Gifford T. A Quantitative assessement of dominance relations among bottlenose dolphins. *Mar. Mamm. Science*, **13**, 70-99 (1997).

4 Owen, E. C., Wells, R. S. & Hofmann, S. (2002). Ranging and association patterns of paired and unpaired adult male Atlantic bottlenose dolphins, *Tursiops truncatus*, in Sarasota, Florida, provide no evidence for alternative male strategies. *Can. J. Zool.* **80**, 2072–2089.

5 Connor, R.C. & Vollmer, N.L. 2009 Sexual coercion in dolphin consortships: a comparison with chimpanzees. In *Sexual coercion in primates: an evolutionary perspective* (eds M.N. Muller & R.W. Wrangham), pp. 218-243. Cambridge, MA: Harvard University Press.

6 Cairns, S. J., & Schwager, S. 1987 A comparison of association indices. *Animal Behaviour*, 3: 1454-1469.

7 Connor, R.C., Heithaus, M.R. & Barré, L.M. Complex structure, alliance stability and mating access in a bottlenose dolphin 'super-alliance'. *Proc. R. Soc. Lond. B*. **268**, 263-267 (2001).

8 Kummer, H. in *Social communication among primates* (ed Altman, S.A.) (University of Chicago Press, 1967).

9 Harcourt, A.H. in *Coalitions and Alliances in Humans and other Animals* (eds Harcourt, A.H. & DeWaal, F.B.M.) (Oxford University Press, 1992).

10 Connor, R.C**.**, Smolker, R.A., & Richards, A.F. in *Coalitions and Alliances in Humans and other Animals* (eds Harcourt, A.H. & DeWaal, F.B.M.) (Oxford University Press, 1992).

11 Connor, R.C. Complex alliance relationships in bottlenose dolphins and a consideration of selective environments for extreme brain size evolution in mammals. *Phil. Trans. Roy. Soc.: Biol. Sci.* **362**, 587-602 (2007).

12 Bissonette A., Franz, M., Schulke O. & Ostner, J. Socioecology, but not cognition, predicts male coalitions across primates. *Behav. Ecol.*, **25**, 794-801 (2014).

13 Smolker, R. A., Richards, A. F., Connor, R. C. & Pepper, J. W. Sex differences in patterns of association among Indian Ocean bottlenose dolphins. *Behaviour* **123**, 38-69 (1992).

14 Connor, R.C., Smolker, R.A. & Bejder, L. Synchrony, social behavior and alliance affiliations in Indian Ocean bottlenose dolphins *(Tursiops aduncus). Anim. Behav.* **72**,1371-1378 (2006).

15 van Schaik C.P., Ancrenaz, M., Borgen, G., Galdikas, B., Knott, C.D, Singleton I, Suzuki A, Utami S.S, Merrill M.Y Orangutan cultures and the evolution of material culture. *Science*. **299**, 102–105 (2003).

16 Whiten, A., Goodall, J., McGrew, W. C., Nishida, T., Reynolds, V., Sugiyama, Y., Tutin, C. E., Wrangham, R. W. & Boesch, C. Cultures in chimpanzee. *Science* **399**, 682–85 (1999).

17 Whitehead, H., & Rendell, L. *The cultural lives of whales and dolphins*. (University of Chicago Press, 2014).

18 Smolker, R.A.; Richards, A.F.; Connor, R.C. & Mann, J. Sponge carrying by dolphins (Delphinidae, *Tursiops* sp.): A foraging specialization involving tool use? *Ethology* **103**, 454-465 (1997).

19 Mann, J., Sargeant, B. L., Watson-Capps, J. J., Gibson, Q. A., Heithaus, M. R., Connor, R. C. & Patterson, E. Why Do Dolphins Carry Sponges? *PLoS One* (2008).

20 Krützen, M., Kreicker, S., MacLeod, C.D., Learmonth, J., Kopps, A.M. & Walsham, P. Cultural transmission of tool use by Indo-Pacific bottlenose dolphins (*Tursiops sp.*) provides access to a novel foraging niche. *Proc. Roy. Soc. London B*. 281 (1784), 20140374 (2014).

21 Sargeant, B., Mann, J., Berggren, P., & Krützen, M. Specialization and development of beach hunting, a rare foraging behavior, by wild bottlenose dolphins (*Tursiops* sp.) *Can. J. Zool.*, **83**, 1400-1410 (2005).

22 Connor, R.C., Heithaus, M.R., Berggren, P. & Miksis, J.L. 'Kerplunking': Surface fluke-splashes during shallow water bottom foraging by bottlenose dolphins. *Mar. Mamm. Sci.*, **16**, 646-653 (2000).

23 Allen S.J., Bejder, L. & Krützen, M, Why do Indo-Pacific bottlenose dolphins (*Tursiops* sp.) carry conch shells (*Turbinella sp*.) in Shark Bay, Western Australia. *Mar. Mamm. Sci.,* **27**, 449-454 (2011).

24 Mann, J., Connor, R.C., Barre, L.M., & Heithaus, M.R. Female reproductive success in bottlenose dolphins (*Tursiops* sp.): life history, habitat, provisioning and group size effects. *Behav. Ecol.* **11**, 210-219 (2000).

25 Connor, R.C., Richards, A.F., Smolker, R.A. & Mann, J. Patterns of female attractiveness in Indian Ocean bottlenose dolphins. *Behaviour* **133** (1-2), 37-69 (1996).

26 Tutin, C.E.G. Mating patterns and reproductive strategies in a community of wild chimpanzees (*Pan troglodytes schweinfurthii*). *Behav. Ecol. Sociobiol.* **6**, 29- 38 (1979).

27 Muller, M.M & Mitani, J.C. Conflict and cooperation in wild chimpanzees. Adv. Stud. Behav. **35**, 275-331 (2005).

28 Randić, S., Connor, R.C., Sherwin, W.B. & Krutzen, M. A novel mammalian social in Indo-Pacific bottlenose dolphins (*Tursiops* sp.): complex male alliances in an open social network. *Proc. R. Soc. Lond. B* **279**, 3083-3090 (2012).
